# Supplementary material for: High-Precision Protein-Tracking With Interferometric Scattering Microscopy
Source: Front Cell Dev Biol. 2020 Nov 3;8:590158. doi: 10.3389/fcell.2020.590158 (PMC7669747; doi:10.3389/fcell.2020.590158)
Supplement: Supplementary file 1 [file Data_Sheet_1.PDF]

# *Supplementary Material*

## 1 FULL-TRAJECTORY MSD

The MSD in which all trajectory points from a single measurement are simultaneously considered is presented in **Fig. S1** for all trajectories presented in this paper. Each panel includes an inset of the respective trajectory from which it is composed. The MSD is computed with ten frames as the smallest time lag and the largest corresponds to 20% of the total trajectory length.

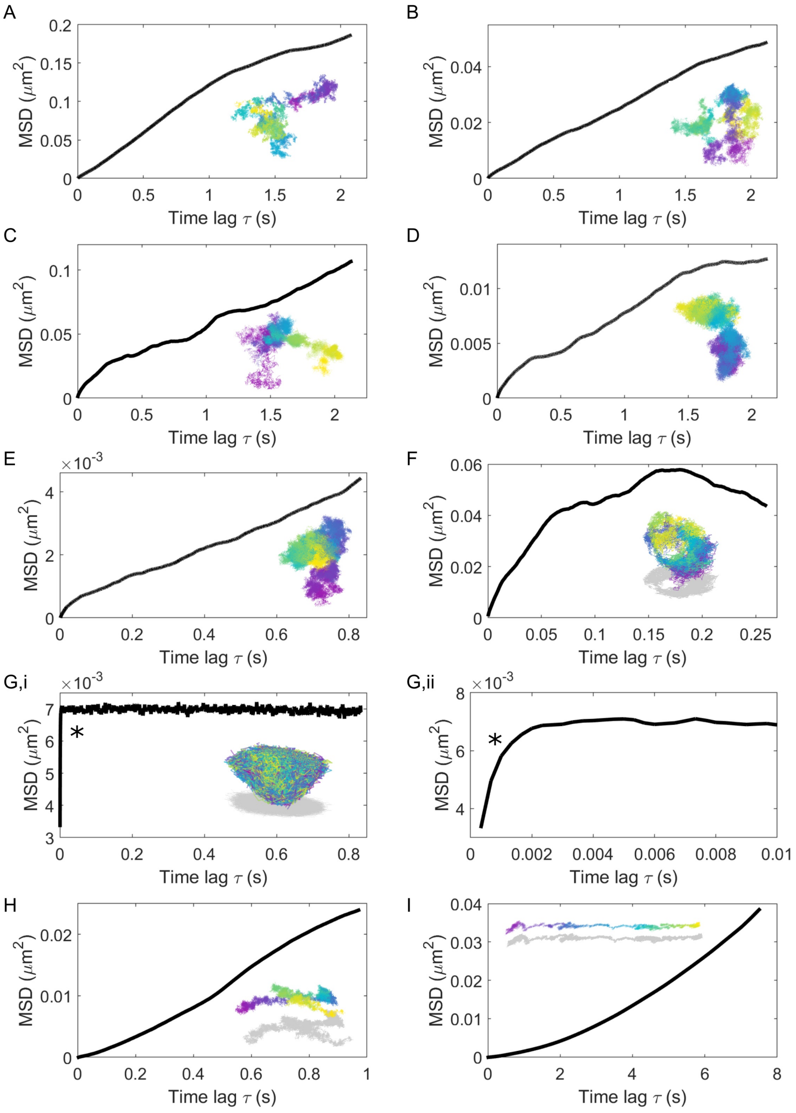


**Figure S1. Full-trajectory MSD for all trajectories in this work. (A-I)** The MSD computed for each trajectory and plotted. Insets show the corresponding trajectory from which each MSD is computed. **(G,i)** The region of the MSD marked with an asterisk is highlighted in (**G,ii**).

## 2 FITTING THE TEMPORAL EXPONENT

To fit the temporal exponent $\alpha_i$, one begins with **Eq. 3**, and recasts accordingly:

$$\log(\mathrm{MSD}_i(\tau) - \sigma_{xy}) = \alpha_i \log(\tau) + \Delta. \tag{S1}$$

In doing so, by taking the logarithm, the desired temporal exponent becomes a linear function of logarithmic time and can be fitted readily with a first order polynomial. The term $\Delta = \log(\Gamma_i)$ is an offset.

**Figure S2** presents an example showing the fitting of $\alpha_i$ from a MSD of a single window. In this example, the window is 1,000 frames long and each frame has a temporal duration of 33 $\mu s$. The green hollow markers show the first 50 time lags. One should ensure the smallest time lags chosen give a MSD larger than the localisation error by which the particle position is known, else the calculation of the temporal exponent will be erroneous (Martin et al. (2002)), which is often typified by a diverging gradient, such as shown in **Fig. S2**. In this work, we chose a lag time of ten frames as the smallest lag, with increments of ten frames up to the longest lag time of 50 frames, shown as solid red markers in **Fig. S2**.

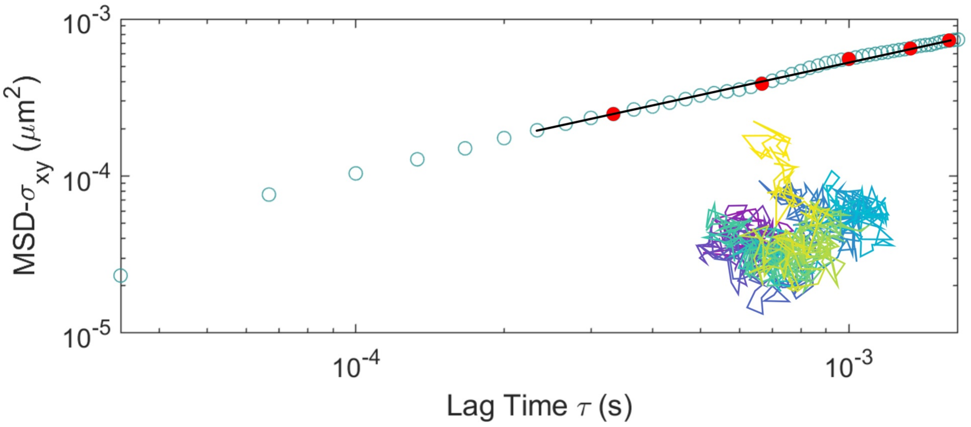


**Figure S2.** Fitting the temporal exponent within a single window, with the window comprising 1,000 trajectory points. Green hollow markers show all available lag times in the window up to a maximum of 50. Red markers represent the chosen sub-set of time lags and their respective MSD to which the fit of the temporal exponent is performed. The black dashed line represents the optimised fit and the inset shows the corresponding trajectory of the windowed data.

An optimised linear fit is performed via MATLAB to the chosen lag times, shown as the black dash line in **Fig. S2**. The 95% confidence interval of the computed fit is used to assign the error to the fitted gradient $\alpha$. In this example $\alpha = 0.69 \pm 0.09$. The fitting process is repeated for all windows within the trajectory to yield $\alpha_i(t)$.

## 3 EFFECT OF WINDOW SIZE ON THE TEMPORAL EXPONENT

The number of frames ($N$) that compose the sliding window for the $\mathrm{MSD}_i$ determines the number of data points over which the sliding MSD can be computed. Increasing the window length reduces the resultant uncertainty in the temporal exponent $\alpha_i$ as a larger number of frames of data is used, but this occurs at the expense of averaging over small-scale variances in $\alpha_i$, as shown in **Fig. S3** for various $N$. Nonetheless, the fitted value of $\alpha_i$ is quite robust against the specific choice of window size. In this work, a window length of 1,000 frames is often found to be an acceptable length (Taylor et al. (2019)).

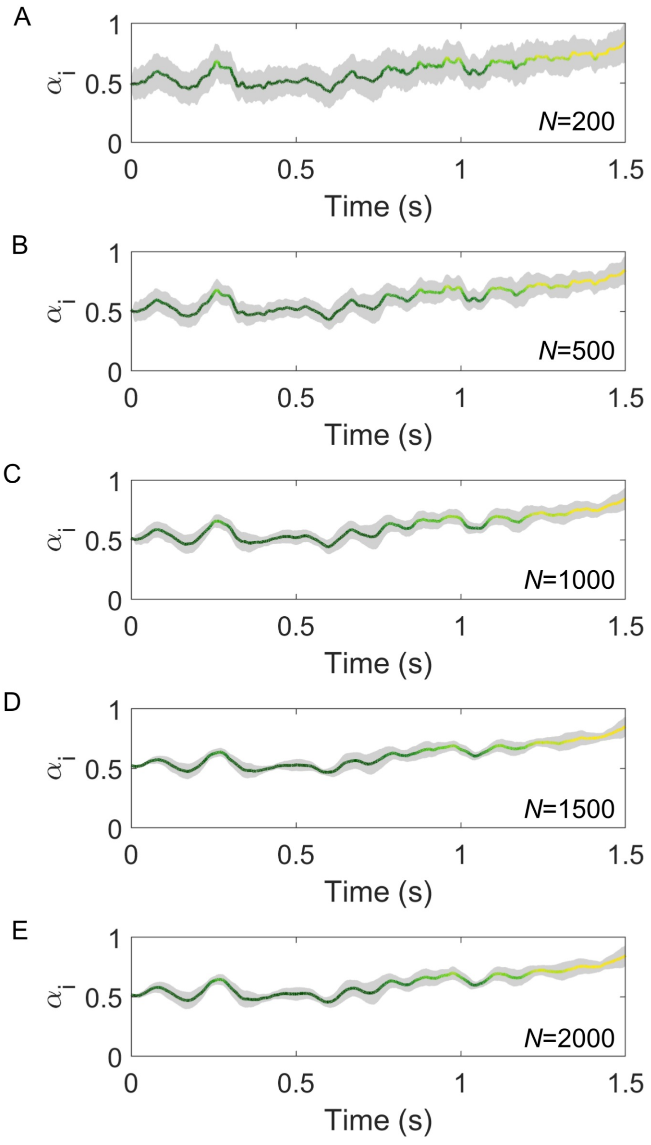


**Figure S3. The effect of increasing the window size on the temporal exponent.** (A-E) The temporal exponent for a portion of a trajectory, as the number of points composing the window, ($N$) is increased.

## REFERENCES

Martin, D. S., Forstner, M. B., and Käs, J. A. (2002). Apparent subdiffusion inherent to single particle tracking. *Biophys. J.* 83, 2109–2117. doi:10.1016/S0006-3495(02)73971-4

Taylor, R. W., Mahmoodabadi, R. G., Rauschenberger, V., Giessl, A., Schambony, A., and Sandoghdar, V. (2019). Interferometric scattering microscopy reveals microsecond nanoscopic protein motion on a live cell membrane. *Nat. Photon.* 13, 480–487. doi:10.1038/s41566-019-0414-6
